# Sequential correction of severe and rigid kyphoscoliosis: a new technical note and preliminary results

**DOI:** 10.1186/s12891-023-06736-9

**Published:** 2023-08-31

**Authors:** Wenbin Hua, Shuai Li, Xiaobo Feng, Kun Wang, Huipeng Yin, Xinghuo Wu, Yukun Zhang, Yong Gao, Li Ling, Cao Yang

**Affiliations:** 1 Department of Orthopaedics, Union Hospital, Tongji Medical College, Huazhong University of Science and Technology, Wuhan, 430022 China; 2 Department of VIP Clinic, Union Hospital, Tongji Medical College, Huazhong University of Science and Technology, Wuhan, 430022 China

**Keywords:** Apical vertebrae, Scoliosis, Kyphoscoliosis, Sequential correction, Spinal osteotomies, Treatment

## Abstract

**Objective:**

The present study is to evaluate the clinical outcomes of the sequential correction of severe and rigid kyphoscoliosis.

**Methods:**

Between January 2014 and December 2020, 27 adults with severe and rigid kyphoscoliosis underwent sequential correction combined with posterior grade 4 or grade 5 spinal osteotomy. Radiological parameters, including the major curve Cobb angle, kyphotic angle, coronal imbalance, and sagittal vertical axis (SVA), were compared. Patient self-reported health-related quality of life (HRQOL) scores were used to evaluate clinical outcomes.

**Results:**

The mean major curve Cobb angle improved from 134.30 ± 13.24° to 44.48 ± 9.34° immediately after surgery and to 46.11 ± 8.94° at the final follow-up. The mean kyphotic angle improved from 112.15 ± 20.28° to 38.63 ± 15.00° immediately after surgery and to 39.85 ± 14.92° at the final follow-up. The mean preoperative major curve Cobb angle of grade 5 spinal osteotomy group was higher than that of grade 4 spinal osteotomy group. Coronal imbalance and SVA slightly improved. The patient self-reported HRQOL scores improved postoperatively and at the final follow-up. Activity, appearance and total scores of the SRS-22 of the grade 5 spinal osteotomy group at the final follow-up were significantly better than those of the grade 4 spinal osteotomy group.

**Conclusions:**

Sequential correction combined with posterior grade 4 or grade 5 spinal osteotomies is an excellent and safe treatment for severe and rigid kyphoscoliosis in adults. Sequential correction combined with posterior grade 5 spinal osteotomies can be used to correct severe and rigid kyphoscoliosis with higher major curve Cobb angle.

## Introduction

The surgical treatment of severe and rigid kyphoscoliosis in adults is challenging, with a high risk of neurological complications. A major curve Cobb angle > 90° and flexibility < 30% are thought to be the criteria for severe and rigid scoliosis [1, 2]. During the surgical correction of severe and rigid kyphoscoliosis, three-column spinal osteotomies may be necessary, including pedicle subtraction osteotomy (PSO), transpedicular wedge resection osteotomy (TWRO, grade 4 spinal osteotomy), vertebral column resection (VCR, grade 5 spinal osteotomy), and multilevel VCR [3–7]. Of the VCRs, circumferential approach VCR and posterior approach VCR (PVCR) are commonly used techniques for correcting severe and rigid spinal deformities [8].

In traditional correction techniques, only two long rods are used to correct severe deformities and maintain global coronal balance and sagittal alignment. Three-column spinal osteotomies and correction procedures are at high risk for intraoperative neurological complications [3–8]. We developed a new technical procedure, sequential correction, to treat severe and rigid kyphoscoliosis. Posterior grade 4 or grade 5 spinal osteotomies are usually combined with sequential correction to achieve better spinal deformity correction. This study aimed to evaluate the clinical outcomes of sequential correction of severe and rigid kyphoscoliosis in adults.

## Methods

### Study design

Between January 2014 and December 2020, 27 adults with severe and rigid kyphoscoliosis underwent sequential correction combined with posterior grade 4 or grade 5 spinal osteotomy. All surgical procedures were performed by the same senior author. According to the selected osteotomy type, the patients were divided into two groups: grade 4 spinal osteotomy (TWRO) and grade 5 spinal osteotomy (PVCR). This study was conducted in accordance with the Declaration of Helsinki and with approval from the Ethics Committee of our Hospital. Written informed consent was provided by all participants.

### Inclusion and exclusion criteria

Inclusion criteria were as follows: 1) age at least 18 years; 2) severe and rigid kyphoscoliosis with a major curve Cobb angle  ≥ 90° and flexibility ≤ 30%. Exclusion Criteria were as follows: 1) major curve Cobb angle < 90° or flexibility > 30%; 2) accompanying infections, tumors, or other lesions; 3) history of spinal surgery.

### Surgical technique

**Fig. 1:**
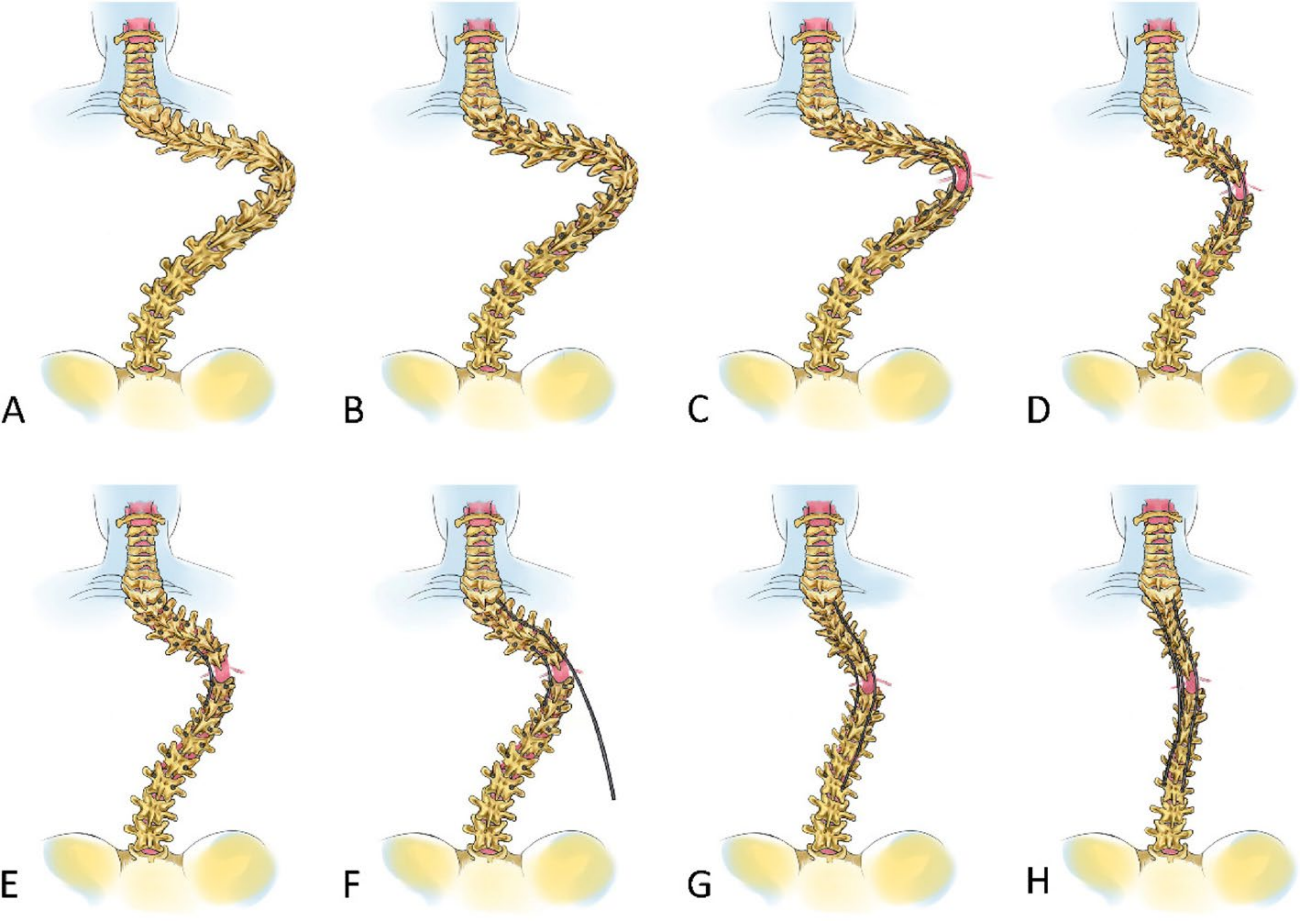
The key steps of sequential correction to treat severe and rigid kyphoscoliosis in adults. **A**, Severe and rigid kyphoscoliosis before correction. **B**, Screw placement and facetectomy. **C**, Grade 4 or grade 5 spinal osteotomy at the apical vertebrae. **D**, Major curve correction at the apical vertebrae. **E**, Correction maintained with short segmental instrumentation. **F**, **G**, Further correction via the rod cantilever technique. H, Integration with appropriate application of long rods

The complete surgical procedures were performed under continuous neuromonitoring, including motor evoked potential and somatosensory evoked potential. The key procedures of sequential correction technique were summarized as following (Fig. 1).

#### Screw placement and facetectomy

Planed implantation of the pedicle screws was performed using a freehand technique [9]. Facetectomy was performed to adequately release the rigid segments to be fused.

#### Asymmetrical three-column osteotomy at the apical vertebrae

C-arm fluoroscopy was used to confirm the apical vertebrae of the kyphoscoliosis, which were then exposed using costotransversectomy. Asymmetrical three-column osteotomies were performed at the apical vertebrae to correct the scoliosis and kyphosis. TWRO or PVCR were performed according to the severity of the deformity.

#### Major curve correction at the apical vertebrae

The osteotomy site was closed step-by-step, partially correcting the scoliosis and kyphosis. Two short segmental rods were used to maintain spinal stability during the closing procedures and prevent intraoperative vertebral subluxation.

#### Correction maintained with short segmental instrumentation

The short segmental rod on the concave side was reserved to maintain correction at the apical vertebrae, whereas the rod on the convex side was removed.

#### Further correction via the rod cantilever technique

A long rod was implanted at the convex side from the upper instrumented vertebra (UIV) to the lower instrumented vertebra (LIV), and the rod cantilever technique was used to further correct the scoliosis and kyphosis, achieving coronal and sagittal balance [10, 11].

#### Integration with appropriate application of long rods

The second long rod was implanted at the concave side from the UIV to the LIV to correct the remaining coronal and sagittal imbalance. After closing the osteotomy sites of the TWRO, the remaining space was filled with autogenous cancellous bone. After closing the osteotomy sites of the PVCR, a titanium cage containing autogenous cancellous bone was applied for vertebral column support, if necessary.

### Data collection

Radiological measurements were obtained preoperatively, postoperatively, and at the final follow-up. Major curve Cobb angles and kyphotic angles were measured. Coronal imbalance was measured as the horizontal distance between the C7 plumb line and center sacral vertical line. The sagittal vertical axis (SVA) was measured as the horizontal distance from the C7 plumb line to the posterior-superior corner of S1.

All patients completed self-reported health-related quality of life (HRQOL) assessments preoperatively, postoperatively, and at the final follow-up. The Oswestry Disability Index (ODI) score and Scoliosis Research Society-22 (SRS-22) questionnaire were obtained.

### Statistical analysis

SPSS 22.0 (IBM Corp., Armonk, NY, USA) statistical analysis software was used to perform the statistical analyses. Variables are presented as mean ± standard deviation. A Kolmogorov–Smirnov test was used to assess the normal distribution of the data. Independent-samples *t* test was used to compare intergroup differences of data normally distributed. Nonparametric tests, including the Mann–Whitney U test and Wilcoxon signed-rank test, were used to compare intergroup differences of data not normally distributed. A *P*-value less than 0.05 is considered as statistically significant.

## Results

### Demographics

The demographic data of patients who underwent sequential correction combined with posterior grade 4 or 5 spinal osteotomy are summarized and compared in Table 1. The demographic data of two groups were of no significant difference.

**Table 1:** Demographics parameters of patients underwent sequential correction combined with TWRO or PVCR

Characteristic	Sequential correction (*n* = 27)	Grade 4 (TWRO) (*n* = 18)	Grade 5 (PVCR) (*n* = 9)	*P*
**Age (yrs)**	30.56 ± 11.18	28.89 ± 10.53	33.89 ± 12.34	0.314
**Sex (Male: Female)**	7:20	4:14	3:6	0.317
**BMI (kg/m**^**2**^**)**	20.46 ± 3.11	19.85 ± 3.40	21.69 ± 2.07	0.095
**Mean follow-up (months)**	37.56 ± 13.88	33.89 ± 12.34	35.33 ± 15.43	0.467
**Scoliosis classification**				
**Idiopathic scoliosis**	14	10	5	0.059
**Congenital scoliosis**	10	6	3	
**Neuromuscular scoliosis**	3	2	1	
**Major curve type**				
**Thoracic**	22	16	6	0.083
**Thoracolumbar/lumbar**	5	2	3	
**Major curve flexibility (%)**	11.46 ± 5.80	10.17 ± 5.63	14.05 ± 5.52	0.056
**Fused levels**	13.67 ± 1.24	13.89 ± 1.32	13.22 ± 0.97	0.153
**Operation time (min)**	507.04 ± 90.69	497.22 ± 94.20	526.67 ± 85.00	0.277
**Estimated blood loss (ml)**	2722.22 ± 547.96	2738.89 ± 561.66	2688.89 ± 551.01	0.828

### Radiologic outcomes

**Fig. 2:**
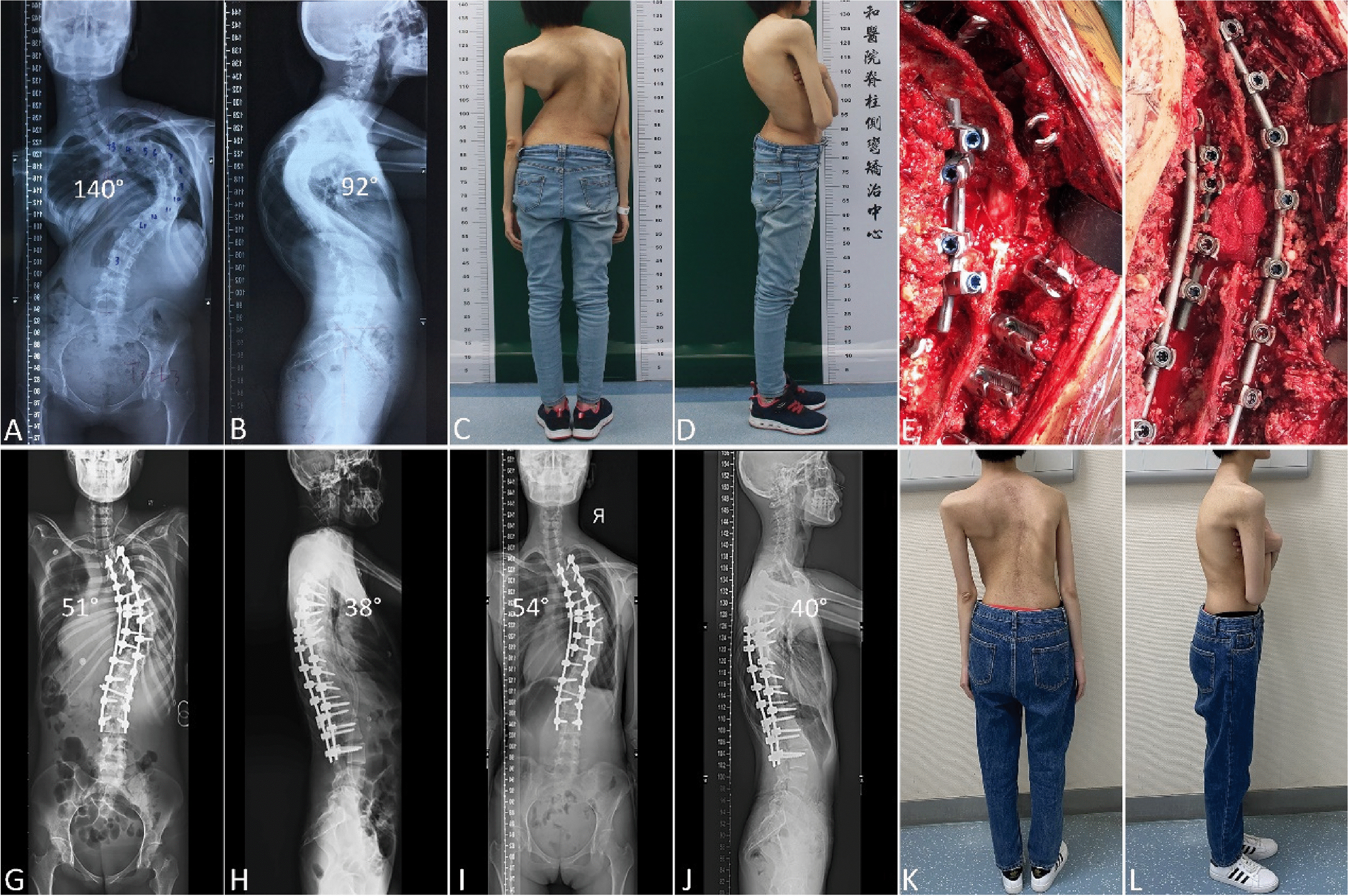
A 25-year-old woman with severe and rigid kyphoscoliosis treated by sequential correction combined with posterior grade 4 spinal osteotomy at the apical vertebrae. **A**, **B**, The coronal major curve angle and the kyphotic angle were 140° and 92° respectively on preoperative anteroposterior and lateral radiographs. **C**, **D**, Preoperative appearance. **E**, Grade 4 spinal osteotomy performed at the apical vertebrae (T9), and apical vertebrae correction maintained with short segmental instrumentation. **F**, Sequential correction completed. **G**, **H**, The coronal major curve angle and the kyphotic angle were 51° and 38° respectively on postoperative anteroposterior and lateral radiographs. **I**, **J**, The coronal major curve angle and the kyphotic angle were 54° and 40° respectively on postoperative anteroposterior and lateral radiographs at 24-month follow-up. **K**, **L**, Postoperative appearance

**Fig. 3:**
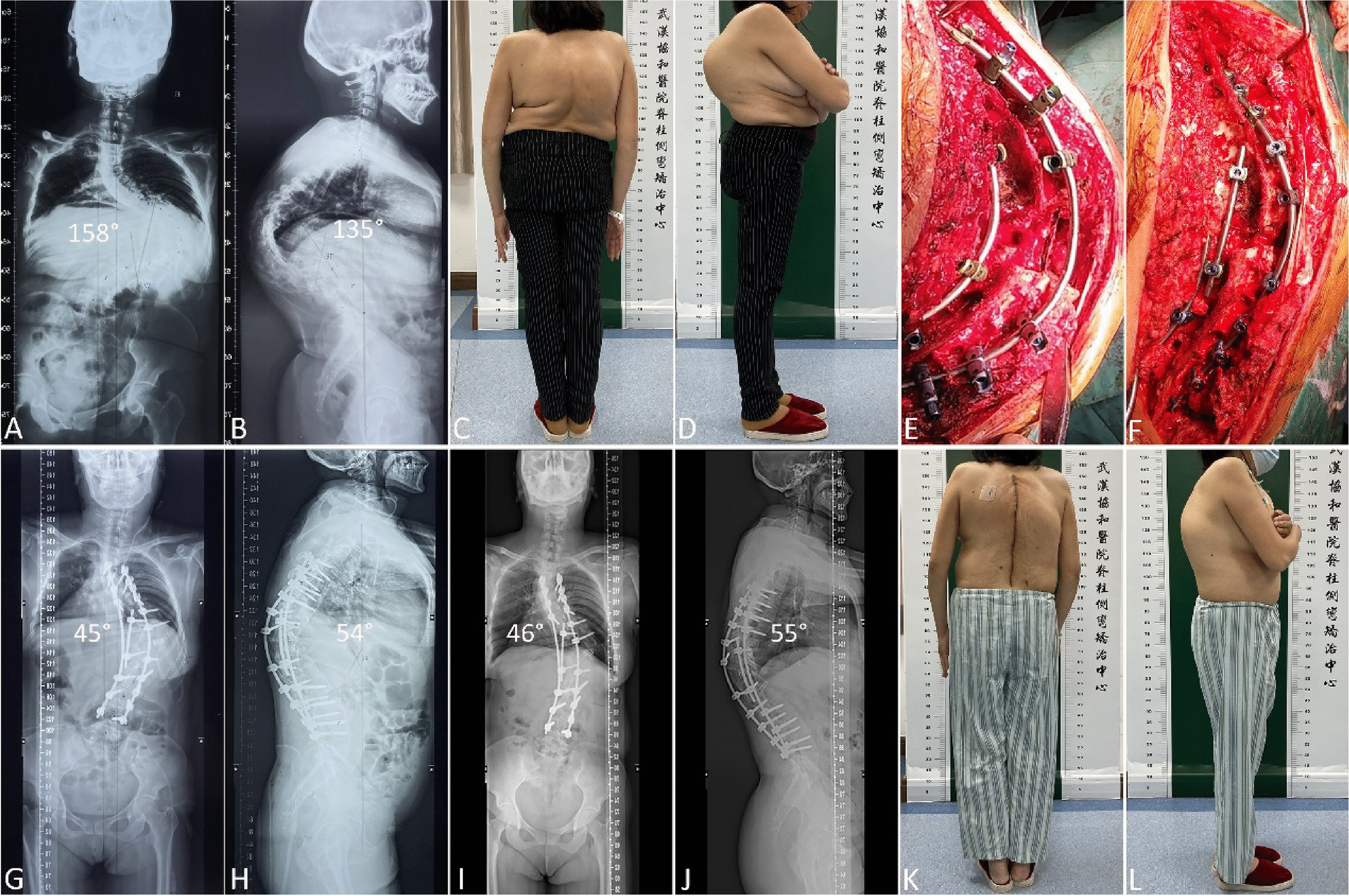
A 48-year-old woman with severe and rigid kyphoscoliosis treated by sequential correction combined with posterior grade 5 spinal osteotomy at the apical vertebrae. **A**, **B**, The coronal major curve angle and the kyphotic angle were 158° and 135° respectively on preoperative anteroposterior and lateral radiographs. **C**, **D**, Preoperative appearance. **E**, Grade 5 spinal osteotomy performed at the apical vertebrae (T12), apical vertebrae correction maintained with short segmental instrumentation. **F**, Sequential correction completed. **G**, **H**, The coronal major curve angle and the kyphotic angle were 45° and 54° respectively on postoperative anteroposterior and lateral radiographs. **I**, **J**, The coronal major curve angle and the kyphotic angle were 46° and 55° respectively on postoperative anteroposterior and lateral radiographs at 24-month follow-up. **K**, **L**, Postoperative appearance

The mean major curve Cobb angles were 134.30 ± 13.24° preoperatively, 44.48 ± 9.34° immediately after surgery, and 46.11 ± 8.94° at the final follow-up. The mean major curve Cobb angle correction (rate) was 89.82° (66.88%) immediately after surgery, and 88.19° (65.67%) at the final follow-up. The mean kyphotic angles were 112.15 ± 20.28° preoperatively, 38.63 ± 15.00° immediately after surgery, and 39.85 ± 14.92° at the final follow-up. The mean kyphotic angles correction was 73.52° immediately after surgery, and 72.30° at the final follow-up. Moreover, the radiographic parameters of the patients who underwent sequential correction combined with posterior grade 4 or 5 spinal osteotomy are summarized and compared in Table 2. The mean preoperative major curve Cobb angle of grade 5 spinal osteotomy group was higher than that of grade 4 spinal osteotomy group. A typical case treated by sequential correction combined with posterior grade 4 spinal osteotomy at the apical vertebrae was listed (Fig. 2). A typical case treated by sequential correction combined with posterior grade 5 spinal osteotomy at the apical vertebrae was listed (Fig. 3).

**Table 2:** Radiographic parameters of the patients underwent sequential correction combined with TWRO or PVCR

		**Sequential correction (*n* = 27)**	**Grade 4 (TWRO) (*n* = 18)**	**Grade 5 (PVCR) (*n* = 9)**	***P***
**Major curve Cobb angle (°)**	**Preop**	134.30 ± 13.24	130.39 ± 11.32	142.11 ± 13.94	**0.039**
	**Post-op**	44.48 ± 9.34*	43.94 ± 9.83*	45.56 ± 8.75*	0.642
	**Final follow-up**	46.11 ± 8.94*	46.00 ± 9.31*	46.33 ± 8.67*	0.959
**Kyphosis angle (°)**	**Preop**	112.15 ± 20.28	108.06 ± 17.62	120.33 ± 23.74	0.303
	**Post-op**	38.63 ± 15.00*	34.22 ± 13.23*	47.44 ± 15.09*	**0.030**
	**Final follow-up**	39.85 ± 14.92*	35.56 ± 13.03*	48.44 ± 15.44*	0.056
**CI (mm)**	**Preop**	32.74 ± 19.35	33.11 ± 15.19	32.00 ± 26.93	0.571
	**Post-op**	29.48 ± 20.34	32.33 ± 20.91	23.78 ± 18.98	0.236
	**Final follow-up**	31.44 ± 23.32	33.61 ± 16.16	27.11 ± 34.37	0.198
**SVA (mm)**	**Preop**	35.33 ± 41.30	35.94 ± 31.56	34.11 ± 58.52	0.326
	**Post-op**	24.78 ± 15.25	24.94 ± 14.69	24.44 ± 17.23	0.959
	**Final follow-up**	22.00 ± 17.33	19.28 ± 16.19	27.44 ± 19.21	0.234

*Preop* preoperation; postop, postoperation, *CI* coronal imbalance, *SVA* sagittal vertical axis

^*^P < 0.001 compared with Preop

### Clinical outcomes

Variability in the self-reported HRQOL of the patients who underwent sequential correction is summarized in Table 3. The mean ODI score improved from 41.11 ± 11.32 preoperatively to 18.37 ± 7.54 at the final follow-up. The SRS-22 questionnaire scores improved significantly at the final follow-up compared to the preoperative data. Activity, appearance and total scores of the SRS-22 of the grade 5 spinal osteotomy group at the final follow-up were significantly better than those of the grade 4 spinal osteotomy group.

**Table 3:** Variance in self-reported health-related quality of life (HRQOL) of the patients underwent sequential correction combined with TWRO or PVCR

		**Sequential correction (*n* = 27)**	**Grade 4 (TWRO) (*n* = 18)**	**Grade 5 (PVCR) (*n* = 9)**	***P***
**ODI**	**Preop**	41.11 ± 11.32	41.00 ± 10.68	41.33 ± 13.19	1.000
	**Final follow-up**	18.37 ± 7.54*	18.00 ± 6.93*	19.11 ± 9.06*	0.574
**SRS-22**					
**Activity**	**Preop**	2.63 ± 0.35	2.64 ± 0.33	2.60 ± 0.40	0.778
	**Final follow-up**	3.90 ± 0.30*	3.82 ± 0.34*	4.04 ± 0.09*	**0.041**
**Pain**	**Preop**	2.67 ± 0.52	2.80 ± 0.56	2.42 ± 0.32	0.091
	**Final follow-up**	4.02 ± 0.34*	3.99 ± 0.32*	4.09 ± 0.40*	1.000
**Appearance**	**Preop**	2.25 ± 0.54	2.07 ± 0.48	2.62 ± 0.45	0.078
	**Final follow-up**	3.44 ± 0.45*	3.31 ± 0.44*	3.69 ± 0.35*	**0.020**
**Mental health**	**Preop**	3.21 ± 0.49	3.19 ± 0.56	3.27 ± 0.32	0.394
	**Final follow-up**	3.93 ± 0.30*	3.91 ± 0.32*	3.98 ± 0.29*	0.228
**Satisfaction**	**Preop**	2.39 ± 0.66	2.33 ± 0.68	2.50 ± 0.61	0.577
	**Final follow-up**	3.91 ± 0.44*	4.00 ± 0.42*	3.72 ± 0.44*	0.317
**Total**	**Preop**	2.64 ± 0.18	2.61 ± 0.21	2.70 ± 0.10	0.260
	**Final follow-up**	3.84 ± 0.15*	3.81 ± 0.16*	3.90 ± 0.12*	**0.043**

*Preop* preoperation; postop, postoperation, *ODI* Oswestry Disability Index, *SRS-22* Scoliosis Research Society 22-item questionnaire, *ODI* 0, no pain, 100, worst conceivable pain, *SRS-22 scale* 5, best; 1, worst

^*^P < 0.001 compared with Preop

### Complications

The complications of the patients who underwent sequential correction are summarized in Table 4. The complication rate of the two groups showed no significant difference. Two patients who underwent sequential correction combined with posterior grade 4 spinal osteotomy had intro-operative neuromonitoring changes. One of them was found with transient neurological complication, and the neurological function recovered after a revision surgery. Another patient was found with transient intro-operative neuromonitoring change and of no neurological complication. One patient who underwent sequential correction combined with posterior grade 5 spinal osteotomy had intro-operative neuromonitoring change. One patient who underwent sequential correction combined with grade 5 spinal osteotomy was treated via a revision surgery for better coronal imbalance correction.

**Table 4:** Complications of the patients underwent sequential correction combined with TWRO or PVCR

Complications	Sequential correction (*n* = 27)	Grade 4 (TWRO) (*n* = 18)	Grade 5 (PVCR) (*n* = 9)	*P*
**Pleural tear (%)**	10 (37.04)	6 (33.33)	4 (44.44)	0.157
**Intro-operative neuromonitoring changes (%)**	2 (7.41)	2 (11.11)	1 (11.11)	0.317
**Transient neurological complications (%)**	1 (3.70)	1 (5.56)	0 (0.00)	0.317
**Permanent neurological complications (%)**	0 (0.00)	0 (0.00)	0 (0.00)	1.000
**Deep infection (%)**	0 (0.00)	0 (0.00)	0 (0.00)	1.000
**Revision surgery (%)**	2 (7.41)	1 (5.56)	1 (11.11)	1.000
**Sum (%)**	15 (51.85)	9 (50.00)	6 (66.67)	0.083

## Discussion

Severe and rigid kyphoscoliosis may affect the thoracic cage, disturbing skeletal, muscular, and diaphragmatic functions, and reducing respiratory system compliance [12]. Patients with severe and rigid kyphoscoliosis may experience restrictive pulmonary and cardiovascular diseases and malnutrition, leading to physical disability or death [12, 13]. Hence, early surgical treatment is recommended to improve pulmonary function and increase life expectancy [12, 14].

Surgical correction of severe and rigid kyphoscoliosis is challenging and associated with a high risk of neurological injury, permanent paralysis, and death [15, 16]. Age, etiology, spinal deformity severity, spinal cord functional classification, osteotomy site, osteotomy type, shortening distance of the osteotomy gap, and correction rate of the deformity angles are common risk factors for neurological complications [17–19]. Moreover, higher osteotomy grades may increase neurological risks [4, 20–22].

Previous studies have used presurgical short-term halo-gravity traction and halo-pelvic traction for treating severe and rigid spinal deformity [12, 23]. Halo-pelvic traction may be more applicable for severe and rigid spinal deformity [24, 25]. However, the related complications of halo-gravity traction and halo-pelvic traction, including nystagmus, pin loosening, infections, neurologic complications, and osteoporosis, should be noted [24, 25]. Anterior release combined with posterior instrumentation may be used to correct severe and rigid spinal deformity [1, 2, 26–29]. Zhou et al. [2] treated 16 patients with severe and rigid idiopathic scoliosis (a mean flexibility of 12.5%) using circumferential approach VCR, the average major curve Cobb angle improved from 99.3° to 32.9° at the immediate postoperative assessment, with a correction of 66.4° (67.0%). Kandwal et al. [29] treated 21 patients with severe and rigid scoliosis (a mean flexibility of 16%) using staged anterior release and posterior instrumentation, the average major curve Cobb angle was corrected from 116.6° to 26.5°, with a correction of 90.1° (77.3%). In our opinion, one-stage posterior surgery may be helpful to reduce the operation time, hospital stay and incidence of perioperative complications.

TWRO or PVCR may be necessary for better correction of severe and rigid kyphoscoliosis. Surgical correction using PVCR at the apical vertebrae of spinal deformities may be the preferred technique for treating severe and rigid spinal deformities, and a major curve Cobb angle correction rate of 48.1–62.4% can be achieved [20, 21, 30–32]. Suk et al. [21] treated 16 patients with severe and rigid scoliosis (a mean flexibility of less than 25%) using PVCR, the average major curve Cobb angle was corrected from 109.0° to 43.1°, with a correction of 65.9° (60.4%). Hamzaoglu et al. [30] treated 102 patients with severe deformity (a mean flexibility of less than 25%) using PVCR, the average major curve Cobb angle was corrected from 102° to 38.3°, with a correction of 63.7° (62.4%). Xie et al. [31] reported 14 patients with rigid kyphoscoliosis via PVCR, the coronal major curve was corrected from 116.6° to 44.9°, with a correction of 71.7° (61.5%). Zhang et al. [32] treated 12 patients with severe and rigid adult idiopathic scoliosis (a mean flexibility of 9.76%) using PVCR, and the average major curve corrected from 108.9° to 56.5°, with a correction of 52.4° (48.1%). The case series in the present study achieved a correction of 89.82° (66.88%), which was better than previous studies. Moreover, sequential correction combined with posterior grade 4 spinal osteotomy achieved a correction of 86.45° (66.30%), and sequential correction combined with posterior grade 5 spinal osteotomy achieved a correction of 96.55° (67.94%). Therefore, sequential correction combined with posterior grade 4 or 5 spinal osteotomy is suitable for the treatment of extremely severe and rigid kyphoscoliosis. Moreover, sequential correction combined with posterior grade 5 spinal osteotomy is suitable for the treatment of severe and rigid kyphoscoliosis with higher major curve Cobb angle.

The major advantage of the sequential correction technique is converting the complex deformity correction into step-by-step procedures, which can be performed more safely. During sequential correction, posterior grade 4 or grade 5 spinal osteotomies were performed at the apical vertebrae to partially correct the major curves. The short segmental rod is greatly important for preventing the displacement of osteotomy sites and maintaining the correction at the apical vertebrae. Then the rod cantilever technique can be used to further correct the major curve and achieve global alignment with fewer complications. Compared to traditional techniques, the sequential correction technique can help restore global coronal alignment. However, the procedures of the sequential correction remain technically demanding and exhausting.

The present study has some limitations. First, there was a small sample size and a short follow-up period. Second, the present study is a single center case series study. Further studies should be conducted to compare the clinical outcomes of sequential correction techniques with traditional techniques for treating severe and rigid kyphoscoliosis. Moreover, multicenter studies with longer follow-up period could be conducted to evaluate the clinical outcomes of the sequential correction of severe and rigid kyphoscoliosis.

## Conclusion

In the present study, a new technique for the sequential correction of severe and rigid kyphoscoliosis was introduced. Sequential correction combined with posterior grade 4 or grade 5 spinal osteotomy is an excellent and safe treatment for severe and rigid kyphoscoliosis in adults. Sequential correction combined with posterior grade 5 spinal osteotomies can be used to correct severe and rigid kyphoscoliosis with higher major curve Cobb angle. Moreover, patients treated by sequential correction combined with posterior grade 5 spinal osteotomies were of better postoperative SRS-22 scores, especially activity, appearance and the total score.

## Data Availability

The data sets supporting the conclusion of this article are included in the manuscript. Upon request, raw data can be provided by the corresponding author.

## Abbreviations

SVA: Sagittal vertical axis
HRQOL: Health-related quality of life
PSO: Pedicle subtraction osteotomy
TWRO: Transpedicular wedge resection osteotomy
VCR: Vertebral column resection
PVCR: Posterior approach vertebral column resection
UIV: Upper instrumented vertebra
LIV: Lower instrumented vertebra
ODI: Oswestry Disability Index
SRS-22: Scoliosis Research Society-22
